# Recursive Subsystems in Aphasia and Alzheimer's Disease: Case Studies in Syntax and Theory of Mind

**DOI:** 10.3389/fpsyg.2016.00405

**Published:** 2016-03-31

**Authors:** Zoltán Bánréti, Ildikó Hoffmann, Veronika Vincze

**Affiliations:** ^1^Department of Psycho-, Neuro- and Socio-linguistics, Research Institute for Linguistics of the Hungarian Academy of Sciences (MTA)Budapest, Hungary; ^2^Department of Hungarian Language, Faculty of Arts, University of SzegedSzeged, Hungary; ^3^Institute of Informatics, University of SzegedSzeged, Hungary; ^4^MTA-SZTE Research Group of Artificial Intelligence, Hungarian Academy of SciencesBudapest, Hungary

**Keywords:** recursive sentence embedding, theory of mind, Broca's aphasia, Alzheimer's disease, compensatory strategy

## Abstract

The relationship between recursive sentence embedding and theory-of-mind (ToM) inference is investigated in three persons with Broca's aphasia, two persons with Wernicke's aphasia, and six persons with mild and moderate Alzheimer's disease (AD). We asked questions of four types about photographs of various real-life situations. Type 4 questions asked participants about intentions, thoughts, or utterances of the characters in the pictures (“What may X be thinking/asking Y to do?”). The expected answers typically involved subordinate clauses introduced by conjunctions or direct quotations of the characters' utterances. Broca's aphasics did not produce answers with recursive sentence embedding. Rather, they projected themselves into the characters' mental states and gave direct answers in the first person singular, with relevant ToM content. We call such replies “situative statements.” Where the question concerned the mental state of the character but did not require an answer with sentence embedding (“What does X hate?”), aphasics gave descriptive answers rather than situative statements. Most replies given by persons with AD to Type 4 questions were grammatical instances of recursive sentence embedding. They also gave a few situative statements but the ToM content of these was irrelevant. In more than one third of their well-formed sentence embeddings, too, they conveyed irrelevant ToM contents. Persons with moderate AD were unable to pass secondary false belief tests. The results reveal double dissociation: Broca's aphasics are unable to access recursive sentence embedding but they can make appropriate ToM inferences; moderate AD persons make the wrong ToM inferences but they are able to access recursive sentence embedding. The double dissociation may be relevant for the nature of the relationship between the two recursive capacities. Broca's aphasics compensated for the lack of recursive sentence embedding by recursive ToM reasoning represented in very simple syntactic forms: they used one recursive subsystem to stand in for another recursive subsystem.

## 1. Introduction

**1.1**. Most linguists use a kind of inductive definition of recursion (Tomalin, 2007; Hulst, 2010b): they define it as the embedding of a constituent in a constituent of the same type in linguistic expressions. Recursion builds complex structures by increasing embedding depth whereas simple iteration yields output structures which do not increase depth (cf. Karlsson, 2010). Watumull et al. (2014) criticize the concept of recursion as articulated in linguistic analysis; they point out that “syntactic embedding is a sufficient, though not necessary, diagnostic of recursion” (p. 1). In the interpretation of our data we will extend the concept of recursion beyond linguistic syntax to the recursive logic of theory-of-mind (ToM) reasoning.

One initial question of our research concerned the particular source of the human faculty of recursion. It is a debated issue whether recursivity, seen as a specific feature of human languageand mind, is a syntactic phenomenon: certain constituents can have the same types of constituents embedded in them, and this operation can be repeatedpotentially unbounded (cf. Hauser et al., 2002; Fitch et al., 2005; Rizzi, 2012), or the source of recursion is semantics or pragmatics: complex propositions can be expressed recursively in human languages (cf. Evans and Levinson, 2009; Everett, 2009), or else recursion is found in general capacities of the human mind (Jackendoff and Pinker, 2005; Pinker and Jackendoff, 2006).

It is also a debated issue what the relationship of those competing alternatives might be. Watumull et al. (2014) argue that recursion is a fundamental linguistic universal. Regarding language (in the wake of Chomsky, 1986) as I-language, they define it as an intensional function that is a mental object, an internal function of all human brains/minds. One of its fundamental features is recursion, i.e., that it may generate infinite sets. They also assign three formal criteria to the capacity of recursion: “the computability of rules generative of non-arbitrary sets; definition by induction enabling the strong generation of increasingly structured expressions; and mathematical induction for the principled (and potentially unbounded) expansion of the generated sets of structures” (p. 6).

Corballis (2011, 2014) argues in favor of the primacy of the recursive operation of the human mind. ToM operations and mental time travel functions (memories of past experiences and imagined future experiences are embedded in present experiences and hence in one another) are operations and functions that involve fundamentally recursive principles and open up infinite possibilities for the mind, at least in principle. In this view, language is based on the recursive nature of ToM or, in a wider sense, on complex mental structures including ToM and recursive structures of mental travel time. Thus, recursive operations are not linguistic ones to begin with: rather, language was adapted to the recursive operation of the mind. The operative tool of recursion is attested in languages (but not to the same extent in all human languages): they are used by language wherever they are “needed,” but it is not a specific property of language itself. Corballis (2011) refers to Grice (1975, 1989)'s theory that it is an essential feature of language (use) that is requires that the speaker should have the intention to change the beliefs in the listener's mind, carried out by making the listener aware of that intention. The interpretation of linguistic statements is based on inferences rather than on explicit decoding. Note that—granted that ToM recursion is crucial for language—in cases where a person has deficits or limitations in his/her ToM operations, we are to expect limitations in his/her linguistic behavior, too, as witnessed by cases of autism (Luyster et al., 2008).

Considering the neural basis of recursion, Friederici et al. (2011) assumed two different computational systems dealing with hierarchical structures: one determined by the cognitive control for complex sequences in non-language domains, and another one (confined to Broca's area) which is able to process hierarchically structured recursive sequences of artificial and natural grammars. The first computational system is less automatic; the second computational system is highly automatic.

Zimmerer and Varley (2010) presented a case study in which syntactic-structural recursion was not available for an agrammatic aphasic participant but his mathematical skills and ToM inferences were unimpaired. Recursive thinking in non-linguistic cognitive domains can be unimpaired in agrammatic aphasia. Siegal and Varley (2006) and Apperly et al. (2006) found intact second order ToM reasoning in severe agrammatism.

On the other hand, with respect to AD, a number of papers discussed deficits of ToM abilities. For instance, Fernandez-Duquet et al. (2008) found that AD persons and persons exhibiting the behavioral variant of fronto-temporal dementia (bFTD) faced similar difficulties in second order false belief tasks (while in other respects they differed from one another). Freedman et al. (2013) demonstrated significant ToM deficit in false belief tests and in visual perspective taking. According to a systematic review by Sandoz et al. (2014), the deficit shows up more markedly in complex ToM tasks like second-order false belief tasks, not independently of changes of ToM reasoning and other cognitive processes in old age. Moreau et al. (2015) demonstrated the presence of ToM deficit in AD persons in tasks requiring realistic communicative interaction, too. Other researchers (e.g., Choong and Doody, 2013) did not find ToM deficit in AD.

**1.2**. The title of the present paper refers to the fact that our case studies in syntax and ToM are concerned with recursive ***sub***systems, not all types of linguistic recursion. We focus on the effect of linguistic limitations in aphasia or Alzheimer's disease (AD) on syntactic recursion as it appears in the embedding of sentences. Of course, syntactic recursion has other instances, too, like the unbounded merge of DPs; and linguistic recursion has other, quite different aspects as well, like the recursion appearing in the hierarchy of prosodic phrases representing syntactic information (cf. Ladd, 1986; Selkirk, 2009; Wagner, 2010). Schreuder et al. (2009) presented an experiment that revealed: edge-marking processes, such as early pitch accent placement (stress shift), are applied recursively to phonological phrases that are embedded in another phonological phrase. Recursive rules were found in phonotactic structures. According to Hulst (2010a), “phonotactic structure displays considerable recursion firstly at the syllable/foot level and, secondly at the word and phrase level” (p. 335). In an event-related brain potential (ERP) study, Honbolygó et al. (2016) investigated prosody-syntax interaction in the case of embedded clauses. The resulting ERP components showed that “sentence prosody has an independent representation characterized by abstract and most probably recursive structure” (p. 32).

The foregrounding of abilities concerning recursive sentence embedding was motivated by the fact that it was in this area that we could best explore and compare the relationship between the linguistic capacity and that for expressing ToM inferences of aphasics and AD participants. The present case studies concentrate on recursive subsystems that are possible in sentence embedding and ToM inferences.

As for using term “*sentence embedding*,” let us take a simple example for embedded clauses introduced by subordinating conjunctions in potentially recursive constructions:

a) [I knew the beautiful girl.]
b) [I knew [that the beautiful girl remembered……]]
c) [I knew [that the beautiful girl remembered [that the boy understood the gesture……]]]

Examples (b) and (c) are recursive constructions. The “……” in the examples express that it is not restricted how many times the formal operation of clause embedding can be executed.

With respect to ToM, we use the term *embedding* in the sense of *perspective embedding*. What we mean by *perspective* is a set of mental states associated with fictive or factual states of affairs (Whalen et al., 2012). In *perspective embedding*, one mental state is embedded within another mental state. In principle, this can go on infinitely; in practice, however, a series of *perspective embeddings* surpassing five instances begins to become incomprehensible (Dunbar, 2000).

*Sentence embedding* and *perspective embedding* are not the same: they constitute two distinct phenomena. Some sentence embeddings do not involve perspective embedding, e.g.,

d) [This was my dog [that chased the cat [that ate the cheese]]].

Perspective embedding often involves increasing syntactic complexity, including cases of clause embedding. However, increasing syntactic complexity does not necessarily mean clause embedding, e.g.,

e) Surprisingly, she was happy/To my surprise, she was happy.

In (each version of) example (e), one mental state is embedded within another mental state, without clause embedding.

Furthermore, perspective embedding can be implemented in very simple constructions, without any increase in syntactic complexity. In our empirical case studies, linguistic data of this kind will also be presented (cf. points **3.3**. and **3.4.)**.

**1.3**. An early forerunner of the present investigation was presented in Bánréti (2010). In that paper, only aphasic persons were studied. Bánréti (2010) observed that, in response to questions concerning pictures that would require recursive sentence embedding in the answers, Broca's aphasic persons responded by simple, short sentences involving ToM inferences, thus avoiding recursive clause embedding. They capitalized on the parallelism between the semantics of ToM embeddings and syntactic-structural embeddings in order to avoid having to produce syntactic-structural recursion, as it were. Bánréti (2010) came to the conclusion that such preference given to ToM answers was based on a selective retention of the linguistic semantic component and on the employment of mental model constructions driven by choice of perspective and shift of perspective, available in Broca's aphasia, too.

In this paper, those conclusions will be reconsidered and developed in the direction suggesting that, in fact, we have to do with compensation strategies that work across the interfaces of various recursive subsystems and make it possible for the application of one recursive subsystem to compensate for the restricted availability of another recursive subsystem. To substantiate that point, we developed a statistical analysis and interpretation of the results of aphasic groups of participants, and we extended the range of participants to six persons with Alzheimer's disease (AD), using the same tests as with the aphasic patients. We will argue that the results show a double dissociation of recursive clause embedding and ToM operations across Broca's aphasics and the AD group. We will analyze the peculiarities of the compensatory operation of the recursive subsystems involved.

**1.4**. The inclusion of AD participants in our investigations was motivated by the fact that a number of studies had shown that AD persons produced patterns of linguistic errors that exhibited partial similarities and overlaps with the linguistic limitations of aphasics. On the other hand, it was also shown that those similarities across behavioral linguistic profiles did not go back to the same anatomical-structural reasons. Underlying the linguistic profile of agrammatic aphasia we find lesions of the anterior part of the brain (e.g., Bastiaanse et al., 2011), while what we see in cases of AD is a gradual progression of microscopic pathological changes that start from the medial temporal lobe and spread in various directions and in various degrees (e.g., Kempler, 2005; Hyman et al., 2012).

Consider a few examples. Fyndanis et al. (2013) see functional causes behind partial similarities across the linguistic profiles of aphasic and AD persons. Injuries of quite different regions of the brain may have similar consequences like the occurrence of limitations in processing resources found in agrammatic aphasia and in AD alike. It is assumed that one of the partially similar consequences of lesions of the anterior part of the brain and of a progression of microscopic pathological changes starting from the medial temporal lobe is a reduction of processing resources. Fyndanis et al. (2013) found dissociation—similar to the linguistic symptoms of agrammatic aphasia (Bastiaanse et al., 2011)—of three functional grammatical categories in native speakers of Greek with mild AD: a relative retention of subject-verb agreement and limitations in tense morphemes on verbs and aspect markers in both sentence production and grammaticality decisions/sentence comprehension. They found that such limitations correlated with the degree to which the given process or feature might be burdensome for the processing/productive systems. For instance, operations with non-interpreted features are “easier” to perform than the production or assessment of verbal tense markers and aspect markers involving interpreted grammatical features, given that the latter two require integration of linguistic and non-linguistic mental representations with one another. On the other hand, Kavé and Levy (2003) did not find such linguistic profiles with native Hebrew mild AD persons: the found the use of both agreement and tense markers fully retained. Altmann et al. (2001) found deficits of closed-class words and morphosyntax in mild AD native speakers of English. Bencini et al. (2011) showed that the syntactic error patterns of AD persons also depend on the syntactic options of their native languages. Whereas, in the case of Italian, a language allowing grammatical covert subjects, AD persons relatively frequently dropped the subject in complex sentences, in the case of English where overt subjects are required the AD subjects did not drop their subjects. The sentence repetition performances (deficits) of two groups with comparable MMSE scores but with different native languages, on the other hand, were rather similar.

Ullman et al. (1997), Cortese et al. (2006), Ullman (2008), and Walenski et al. (2009) point out that AD persons' linguistic limitations are mainly revealed by their erroneously producing regular forms for lexical items with irregular morphology, primarily due to limitations of the system of declarative memory, while syntactic processes are retained due to their unimpaired system of procedural memory. These data show similarities with the linguistic profile of posterior aphasia.

Other studies have shown that in AD the retention of syntax is coupled with semantic deficit, among other things, in processing sentences containing non-agentive psych verbs whose thematic structure does not follow the standard thematic hierarchy. If thematic roles are to be assigned in the lack of an Agent, deviations from canonical argument realization yield limited performance (e.g., Manouilidou and de Almeida, 2009; Manouilidou et al., 2009). Similar comprehension deficits were also reported in Broca's aphasia (e.g., Finocchiaro, 2002; Piñango, 2000, 2006).

Szatlóczki et al. (2015) found phonetic limitations in spontaneous speech production already in early AD stages, along with semantic-pragmatic limitations. Laurent and Noiret (2015) argue that AD limitations of the visual perceptual and processing system may affect higher level cognitive functions, including performance in linguistic production tasks involving a visual component.

Various studies have found a wide variety of sometimes contradictory patterns of linguistic deficits and limitations in AD. But as far as we know, it has not been investigated so far how the functional linguistic limitations sketched above affect the operations of recursive clause embedding in cases of mild and moderate AD. This is what motivated the involvement of AD participants in our experiments.

The experiments to be presented involved three persons with Broca's aphasia, two persons with Wernicke's aphasia, six persons with mild and moderate AD and 20+6 healthy control participants.

## 2. Experiment #1: Aphasic participants

### 2.1. Participants

All aphasic participants had a left unilateral brain lesion. Participants were assigned to aphasia types on the basis of CT results and the Western Aphasia Battery (WAB) tests (Kertesz, 1982). WAB test was adapted to Hungarian by Osmánné (1991).

Information about the aphasic participants in relation to demographical and lesion data, and the type of aphasia is provided in Table 1.

**Table 1:** **Data of the aphasic participants**.

**Participant**	**P.I**.	**K.M**.	**S.H**.	**S.T**.	**K.J**.
Age	32	67	29	45	32
Education	12	12	11	11	11
Sex	M	F	F	M	M
Handed	Right	Right	Right	Right	Right
Lesion	Ischaemic stroke on the area of the left arteria cerebri media	Ischaemic stroke on the area of the left arteria cerebri media	Left fronto-temporo-parietal lesion, middle cerebral artery infarction	Oedema of left parietal cortex	Left temporal haematoma of a traumatic origin
Time post-stroke (months)	11	12	10	12	10
Aphasia Quotient (AQ) of the WAB	40.0	48.0	52.6	48.2	47.8
Diagnosis	Moderate severe Broca's aphasia	Moderate severe Broca's aphasia	Moderate Broca's aphasia	Moderate severe Wernicke's aphasia	Moderate severe Wernicke's aphasia

#### Control group

The healthy control participants, matched in age to the aphasic participants, are shown in Table 2.

Table 2**Data of the control group**.

**Table:** 

**Participant**	**B.I**.	**L.B**.	**D.A**.	**Sz.I**.	**P.M**.	**P.A**.	**F.Gy**.	**N.A**.	**F.Z**.	**K.J**.
Age	54	28	22	62	28	23	71	22	42	71
Education	11	12	16	16	17	15	16	15	16	8
Sex	F	F	F	M	M	F	M	M	M	F
Handed	Right	Right	Right	Right	Right	Right	Right	Right	Right	Right

**Table:** 

**Participant**	**G.O**.	**R.T**.	**M.Zs**.	**T.B**.	**S.H**.		**K.B**.	**SZ.E**.	**SZ.G**.	**Te.J**.	**T.J**.	**Mean**
Age	26	40	55	56	28		38	66	37	59	38	43.3
Education	16	16	16	12	12		16	16	14	16	14	14.5
Sex	F	F	F	F	F		F	F	M	F	F	–
Handed	Right	Right	Right	Right	Right		Right	Right	Right	Right	Right	–
												

All participants were identified as right handed, native Hungarian speakers.

### 2.2. Materials and methods

Photographs depicting simple situations of everyday life were selected from the Everyday Life Activities test (Stark, 1998). The photographs were presented in a 19-inch computer screen. Two hundred and eight different pictures were used. Twenty of these were used in a pre-test practice phase. The test was administered in three sessions (three subtests). At least 10 days elapsed between sessions. Each particular picture was used only once throughout the full test procedure. The test was self-paced; the pictures were presented one by one when the participant pressed the space key. Three seconds after the picture appeared on the screen, the related question was heard from a sound file. The participant her/himself decided on the amount of time devoted to each answer. When the answer was completed or when the participant gave up answering, s/he pressed the space key again. Then, a blank gray screen appeared. No evaluation or comment was given on the answers during the test. The space key being pressed again, the next picture appeared, and 3 s later, the next question was heard. The structural types of questions (see below) varied randomly within each session/subtest. Participants were allowed to give one or several answers to each question or they could indicate they had no answer by saying “I don't know.” All answers were analyzed in terms of content, relation to the structure of the given question, grammaticality, and the syntactic category of the construction used. The photographs depicted everyday situation and were accompanied by questions of various grammatical types. The types of questions involved were as follows:

Type **1**: *What is X doing in the picture?*

The question does not require that any of its own constituents should be involved in the structure of the answer.

Type **2**: *What does X hate/like/want/ …every afternoon/in her office etc*.?

The answer should be structurally linked to the question and involve:

- a subordinate clause in direct object role, introduced by a potentially recursive operation and signaled by a subordinating conjunction, or
- the verb of the question and its infinitival direct object, or
- a definite noun phrase in the accusative.

Type **3**:*What may be the most entertaining/unpleasant/urgent thing for X to do?*

The answer should be structurally linked to the question and involve:

- a subordinate clause in subject role, introduced by a potentially recursive operation and signaled by a subordinating conjunction, or
- a bare infinitive subject, or
- a definite noun phrase in the nominative.

Type **4**: *What may X be saying/thinking/reminding Y of/asking Y to do* etc.?

The structurally linked answer is

- an embedded clause introduced by a subordinating conjunction, a potentially recursive construction,
- a DP/NP in the accusative/or marked for other cases.

It is important to note that Hungarian Verbs like *say, think, remind, ask* do **not** obligatorily have sentential complements. These Verbs can have simple DP, NP complements as well. Cf.: ő* mondta a friss híreket* “she presentedthe latest news”; *gondolt a lakás árára is* “he thought of the price of the flat as well”; *emlékeztette a feladatára* “she reminded him of his duty”; *segítséget kért* “she asked for help”; *megkérdezte a jó irányt*” he asked for the right direction,” etc.

Type 1 questions did not restrict the structure of the answer in any way. Type 2 and Type 3 questions allowed for recursive and non-recursive answers alike. Type 4 questions could be answered in a structurally linked way by using embedded clause, introduced recursively.

The research has been approved by the Research Ethics Committee of the Research Institute for Linguistics of the Hungarian Academy of Sciences, Budapest, Hungary (30/7/2014). All participants provided consent before participating in the test sessions.

Our results will be analyzed by using statistical significance tests—χ^2^-tests in order to investigate whether there are significant differences in the distribution of grammatical categories between two groups (i.e., a group of participants with impairment and a group of healthy controls)-, and we will provide the level of significance and effect size (in terms of Cramer's V). Analyses were performed using IBM SPSS Statistics software, version 22.0.0.0.

## 3. Results

### 3.1. An overview of the patterns of responses by aphasic participants

Responses given by the five aphasic and 20 healthy control participants have been classified in terms of whether they were structurally linked to the questions and were or were not grammatical.

**Figure 1:**
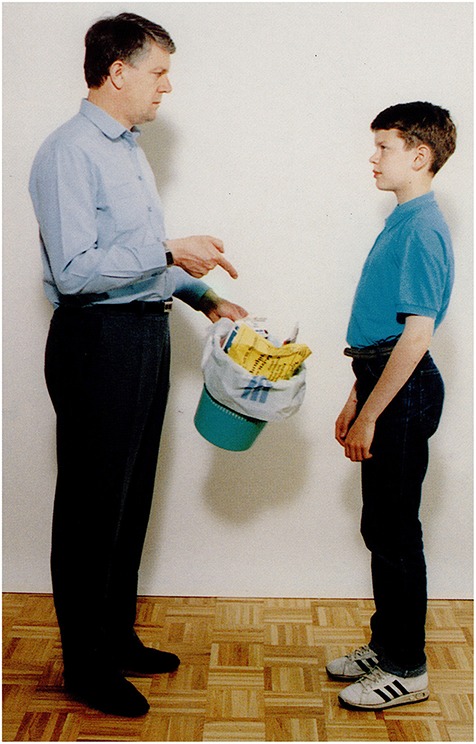
**A man orders a boy to take the garbage out (Stark, 1998)**.

As an example for a response that is included in “all responses” but is **not** structurally linked to the question, see Figure 1.

The picture: A man orders a boy to take the garbage out.

Question: *Mit mondhat az apa a fiúnak?*

             ‘What may the father be saying to the son?’

P.I's answer: *hát a fiú-t,…a fiú-t*,

             well the boy-acc…the boy-acc

A possible recursive construction:

             *Hogy vigye ki a szemetet*.

             ‘That he should take the garbage out.’

In what follows, we present the number of structurally linked answers and all responses given by the participants (Broca's aphasics: **P.I**., **K.M**., and **S.H**., Wernicke's aphasics: *K.J*. and *S.T*.) See Table 3.

**Table 3:** **Numbers and percentages of structurally linked answers compared to all responses for Type 1, 2, 3, and 4 questions per participant**.

**Participant**	***S.T*.**	***K.J*.**	**P.I**.	**K.M**.	**S.H**.
Type 1 question:	61.11%	75.9%	50.54%	71.03%	57.62%
all responses**/**structurally linked answers	72**/**44	83/63	186/94	107/76	151/87
Type 2 question:	53.95%	38.46%	41.63%	61.48%	39.44%
all responses**/**structurally linked answers	76/41	182/70	209/87	122/75	213/84
Type 3 question:	61.76%	59.09%	38.84%	45.18%	43.33%
all responses**/**structurally linked answers	68/42	132/78	224/87	166/75	180/78
Type 4 question:	83.72%	50%	59.52%	66.67%	54.9%
all responses**/**structurally linked answers	43/36	80/40	126/75	90/60	153/84

*S.T. and K.J. = Wernicke's aphasic participants; P.I., K.M., and S.H. = Broca's aphasic participants*.

A summary of the number of structurally linked answers and structurally not linked answers in the types of aphasia and in the control group is given in Table 4 below.

**Table 4:** **Numbers and percentages of structurally linked answers and structurally not linked answers compared to all responses in Type 1, 2, 3, and 4 questions, in the various types of aphasia and in the control group**.

**Participant**	**Wernicke's aphasics**	**Broca's aphasics**	**Control group**
Type 1 question, structurally linked answers	69.03%	57.88%	100%
	107	257	1064
Type 1 question, structurally **not** linked answers	30.97%	42.12%	0%
	48	187	0
Type 2 question, structurally linked answers	43.02%	45.22%	100%
	111	246	1062
Type 2 question, structurally **not** linked answers	56.98%	54.78%	0%
	147	298	0
Type 3 question, structurally linked answers	60%	42.11%	100%
	120	240	1085
Type 3 question, structurally **not** linked answers	40%	57.89%	0%
	80	330	0
Type 4 question, structurally linked answers	61.79%	59.35%	100%
	76	219	982
Type 4 question, structurally **not** linked answers	38.21%	40.65%	0%
	47	150	0

As Table 4 showes, the ratio of structurally linked answers to all responses decreased from the most simple Type 1 (*What is X doing?*) to Type 2 and Type 3 (*What does X want?* and *What is the most entertaining for X?*, respectively). In the case of S.T., the decrease was partial. With Type 4 questions (*What may X be saying/thinking/reminding Y of/asking Y to do?*), the ratio of structurally linked answers increased and for two participants (P.I., S.T.) it turned out to be better than with Type 1 questions and for three participants (K.J., K.M., S.H.), it was almost as good.

We found that the number of structurally linked answers differs significantly for both Broca's and Wernicke's aphasics, as compared to the control group, in the case of all question types, that is, aphasics gave fewer structurally linked answers than the control group did:

Broca vs. control, Type 1 questions: $\chi_{\left(2,\;N=1508\right)}^{2}=511.56$, *p* < 0.001, *V* = 0.58;

Wernicke vs. control, Type 1 questions: $\chi_{\left(2,\;N=1219\right)}^{2}=343$, *p* < 0.001, *V* = 0.53;

Broca vs. control, Type 2 questions: $\chi_{\left(2,\;N=1606\right)}^{2}=714.3$, *p* < 0.001, *V* = 0.67;

Wernicke vs. control, Type 2 questions: $\chi_{\left(2,\;N=1219\right)}^{2}=1002.77$, *p* < 0.001, *V* = 0.91;

Broca vs. control, Type 3 questions: $\chi_{\left(2,\;N=1655\right)}^{2}=784.6$, *p* < 0.001, *V* = 0.69;

Wernicke vs. control, Type 3 questions: $\chi_{\left(2,\;N=1285\right)}^{2}=462.8$, *p* < 0.001, *V* = 0.6;

Broca vs. control, Type 4 questions: $\chi_{\left(2,\;N=1351\right)}^{2}=449.04$, *p* < 0.001, *V* = 0.58;

Wernicke vs. control, Type 4 questions: $\chi_{\left(2,\;N=1105\right)}^{2}=391.9$, *p* < 0.001, *V* = 0.6;

### 3.2. Responses to type 1–3 questions

In what follows, we will base our statistical analyses on grammatically well-formed responses as a subset of structurally linked responses. On the other hand, tables presenting percentages will also show those of structurally linked responses, in addition to those of grammatically well-formed ones.

For Type 1 questions (*What is X doing in the picture?*), most structurally linked and grammatical answers contained Verb Phrases. Only two participants produced a few sentences and accusative Noun Phrases. Participants did not produce recursive syntactic structures at all. The distribution of grammatical categories used in the grammatical responses differs significantly for Broca and Wernicke aphasics [χ^2^-test, $\chi_{\left(2,\;N=283\right)}^{2}=8.72$, *p* < 0.05, *V* = 0.18].

For Type 2 questions (*What does X hate/like/want/ …every afternoon/in her office?)*, most answers involved non-recursive infinitives or accusative noun phrases. Recursive sentence embedding was avoided. The distribution of grammatical categories used in the grammatical responses differs significantly for Broca and Wernicke aphasics for Type 2 questions as well [χ^2^-test, $\chi_{\left(2,\;N=303\right)}^{2}$ = 32.09, *p* < 0.001, *V* = 0.33].

For Type 3 questions (*What may be the most entertaining/unpleasant/urgent thing for X to do?)* most answers involved NP subjects or Infinitives. Participants avoided giving recursive answers as a rule; the few clausal answers produced by S.T., P.I., and K.M. failed to involve a subordinating conjunction. The clausal answers produced by S.H. contained subordinating conjunctions were structurally linked to the questions but were not grammatical; only very few of them were structurally linked and grammatical as well. Comparing Broca and Wernicke aphasics, the distribution of grammatical categories used in the grammatical responses differs significantly for Type 3 questions [χ^2^-test, $\chi_{\left(2,\;N=251\right)}^{2}$ = 21.12, *p* < 0.001, *V* = 0.29]. See Table 5.

**Table 5:** **Numbers and percentages of grammatical responses (in brackets: those of all structurally linked responses) in the various grammatical categories for Type 1, 2, and 3 questions per participant**.

**Participant**	***S.T*.**	***K.J*.**	**P.I**.	**K.M**.	**S.H**.
Responses to TYPE **1** questions	*n* = (44) 36	*n* = (63) 45	*n* = (94) 67	*n* = (76) 68	*n* = (87) 67
Verb	(36.36% 16)	(44.44% 28)	(39.36% 47)	(59.21% 45)	(26.44%) 34.33%
	41.67 % 15	48.89% 22	55.22% 37	61.76% 42	23
Verb Phrase	(63.34% 28)	(34.92% 22)	(42.55% 40)	(39.47% 30)	(16.09%) 20.9%
	58.33% 21	31.11% 14	37.31% 25	36.76% 25	14
Noun Phrase+accusative case ending	**–**	(14.29% 9)	(7.45% 7) 7.47%	(1.32%) 1.47%	**–**
		11.11% 5	5	1	
**S**imple sentence	**–**	(6.35%) 8.89%	**–**	**–**	(57.47% 50)
		4			44.77% 30
Responses to TYPE **2** questions	*n* = (41) 41	*n* = (70) 50	*n* = (87) 78	*n* = (75) 64	*n* = (84) 70
Infinitive Phrase	(68.29%) 68.29%	(48.57% 34)	(63.22% 55)	(93.33% 67)	(55.95% 47)
	28	52% 26	58.97% 46	92.19% 59	52.86% 37
Noun Phrase +accusative case ending	(7.32%) 7.32%	(40% 28)	(5.75%) 6.41%	(8% 6)	(13.1%) 15.71%
	3	42% 21	5	4.67% 3	11
Verb Phrase	(14.63%) 14.63%	(11.43% 8)	(31.03%) 34.62%	(2.67%) 3.12%	(13.1% 11)
	6	6% 3	27	2	10% 7
Simple sentence	**–**	**–**	**–**	**–**	(17.86%) 21.43% 15
Deixis	(9.76%) 9.76%	**–**	**–**	**–**	**–**
	4				
Responses to TYPE **3** questions	*n* = (42) 27	*n* = (78) 37	*n* = (87) 79	*n* = (75) 66	*n* = (78) 42
Infinitive Phrase	(54.76% 23)	(41.03% 32)	(58.62% 51)	(88% 66)	(23.08% 18)
	59.26% 16	64.86% 24	54.43% 43	96.97% 64	35.71% 15
NP-nominative case	(30.95% 13)	(58.97% 46)	**–**	(9.33% 7)	(23.08%) 42.86%
	22.22% 6	35.14% 13		0% 0	18
Simple sentence	(7.14%) 11.11%	**–**	(25.29%) 27.85%	(2.67%) 3.03%	**–**
	3		22	2	
Verb Phrase	**–**	**–**	(16.09%) 17.72%	**–**	**–**
			14		
Subordinating conjunction + descriptive clause	(7.14% 3) 7.4% 2	**–**	**–**	**–**	(53.84% 42) 21.43% 9

### 3.3. Responses to type 4 questions

Responses to Type 4 questions (*What may X be saying/thinking/reminding Y of/asking Y to do?*), may require recursively embedded clauses as answers. As Tables 3, 4 showed, the performance of two of the participants (S.T., P.I.) involving structurally linked answers actually turned out to be better than with Type 1 questions (*What is X doing?*); for three participants (K.J., K.M., S.H.), it was almost as good. This result flies in the face of the expectation that building recursive structures should be more difficult than building non-recursive ones.

Wernicke's aphasics (S.T., K.J.) produced some conjunction-initial descriptive clauses (*that*-clauses) and some simple clauses involving the subjunctive (i.e., the mood directly indicating subordination).

Two of the Broca's aphasics (P.I., K.M.) did not give embedded clauses at all, and one participant(S.H.) produced very few. One participant (K.M.) did produce simple clauses involving the subjunctive, a verbmood indicating potential subordination without *that*-type conjunction. However, most of the structurally linked and grammatical answers produced by Broca's aphasics, as well as the rest of the answers given by Wernicke's aphasics, were rather peculiar: they produced statements that assumed the point of view of one of the characters seen in the picture, rather than being purely descriptive. These participants answered the question as if they were in the “mental state” of the characters or as if they quoted their words in the first person. These answers will be referred to as “situative statements with ‘ToM’ (theory of mind)type reasoning.” In them, the Verb was inflected in the first, rather than the third, person singular (or second person singular, with reference to the partner in the situation shown in the picture), their meanings differed sharply from descriptive statements, as they **directly** represented the thought or statement of the character they “cited.” The participants imagined themselves, as it were, to be in the psychological state of the person in the picture, they mentally simulated and analyzed it, using their own minds as models, and selected their conclusions from among the states thus generated. The application of situative statements made it possible for them to use very brief and simple linguistic structures. Most of the situative statements did not involve a subordinating conjunction, but represented “ToM” type reasoning in the form of simple clauses (It was only Wernicke's aphasic K.J. who produced more *that*-conjunction + descriptive clause answers and in whose case simple situative statements did occur as a minority solution, either).

### 3.4. Examples for the types of responses to type 4 questions

**Figure 2:**
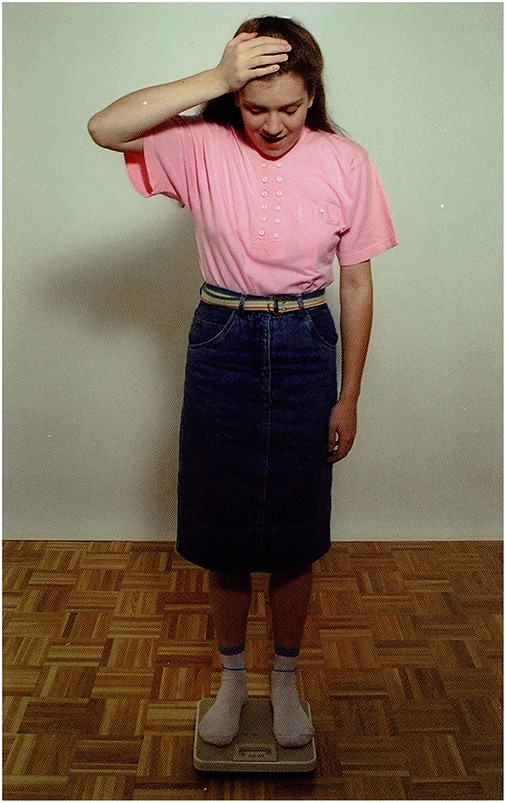
**A girl is standing on bathroom scales (Stark, 1998)**.

**3.4.1**. Situative statement with “ToM” reasoning by Broca's aphasic participants. See Figure 2.

The picture: A girl is standing on bathroom scales.

Question: *Mire gondolhat a lány?*

             ‘What may the girl be thinking of?’

P.I.'s answer: Ú*risten! Ennyi kiló!*

             ‘O my God! So much!’

A possible recursive construction:

             *(Ő) arra gondol, hogy hány kiló lehet*

             ‘She is thinking of how much she may weigh.’

**Figure 3:**
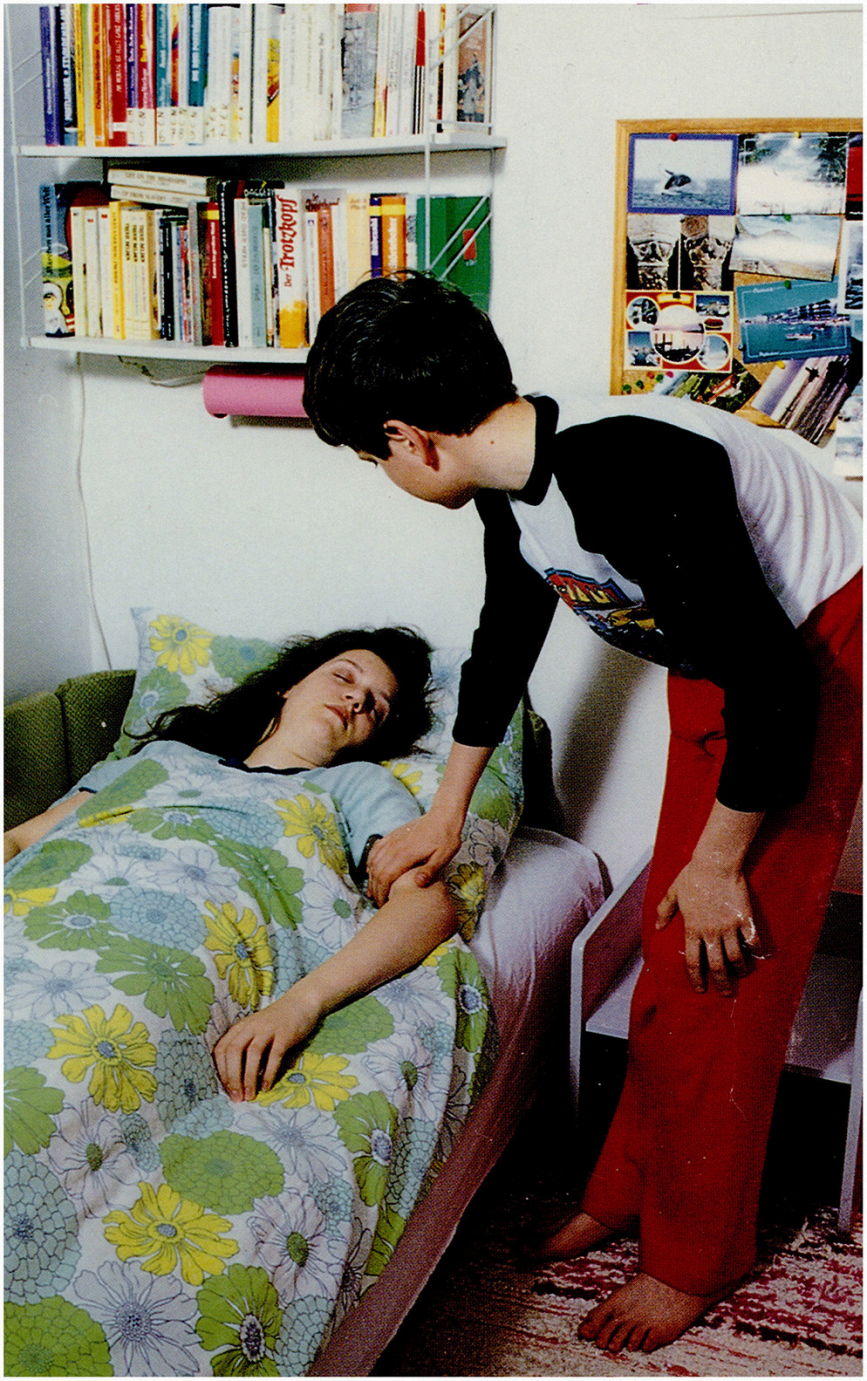
**A boy is waking up a girl (Stark, 1998)**.

**3.4.2**. Subordinating conjunction *that* introduces multiple (second order) ‘ToM’ type reasoning in response by participant S.H. The response contains the first and second person singular features. See Figure 3.

The picture: A boy is waking up a girl.

Question: *Vajon mit mond a fiú a lánynak?*

             ‘What may the boy be saying to the girl?’

S.H.'s answer:

             *Hogy…*. ***te*** *miért vagy szomorú, úgy érze****d****, fáj a feje****m***, *például?*

             ‘That….why are **you** sad, **you** can feel that **I** have a headache, for example?’

A possible recursive construction:

             *A fiú kérdezi a lányt, hogy miért szomorú*.

                 ‘The boy is asking the girl (that) why she is sad.’

**Figure 4:**
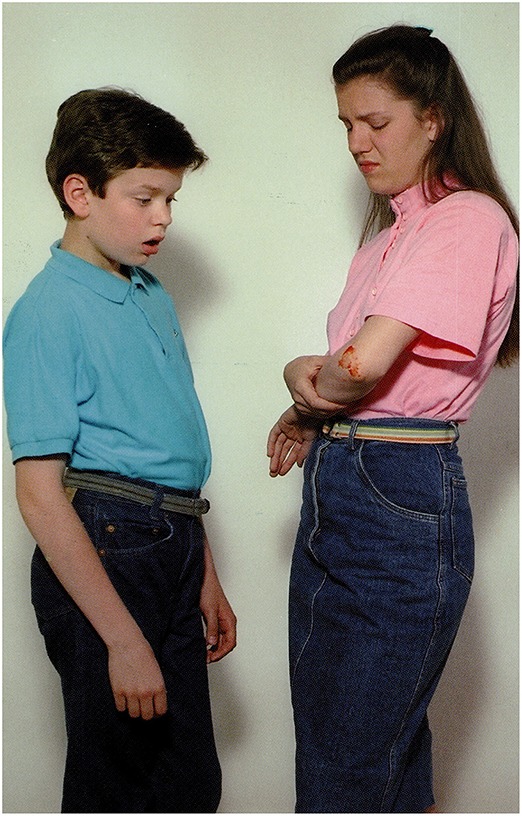
**A girls is showing her scar to a boy (Stark, 1998)**.

**3.4.3**. ‘ToM’ type reasoning by Wernicke's aphasic participant, see Figure 4:

The picture:A girl is showing her scar to a boy.

Question: *Vajon mire gondol a fiú?*

             ‘What may the boy be thinking of?’

S.T.'s answer: *Mindjárt rosszul leszek!*

             ‘I'm going to be sick.’

A possible recursive construction:

             *(Ő) arra gondol, hogy mindjárt rosszul lesz…*.

                   ‘He thinks (that) he is going to be sick….’

**Figure 5:**
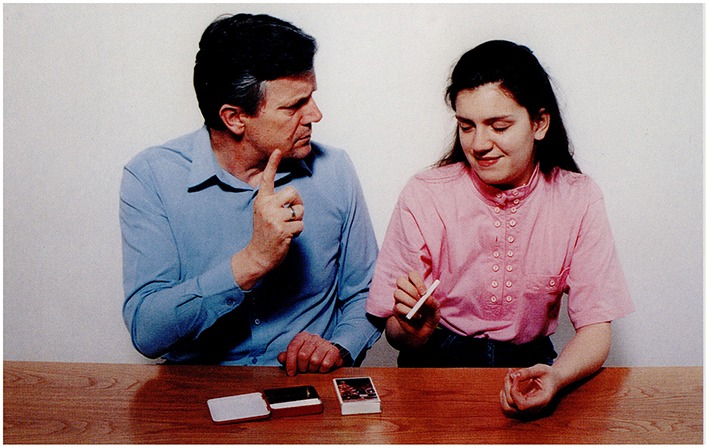
**A father warns his daughter that she should not smoke (Stark, 1998)**.

**3.4.4**. Syntactic-structural recursion in a response by a Wernicke's aphasic participant, see Figure 5.

The picture: A father warns his daughter that she should not smoke

Question: *Mire figyelmeztetheti az apa a lányt?*

             ‘What may the father be warning his daughter about?’

K.J.'s answer: *Hogy nem szabad cigarettázni, hogy az veszélyes*.

             ‘That she should not smoke, that it is dangerous.’

It is important to note that Type 2 and Type 3 questions also required inferences on the mental state of the characters to be drawn from the pictures but participants did not produce situative statements in their responses to Type 2 and Type 3 questions.

**3.5**. Table 6 below shows the number of structurally linked responses (outside the brackets: that of grammatical responses) in the various grammatical categories for Type 4 questions. The number of situative statements containing ToM reasoning in answers to Type 4 questions is also given. These responses were supposed to involve recursive sentence embeddings but they contain “ToM” inferences instead. See Table 6.

**Table 6:** **Numbers and percentages of grammatical responses (in brackets: those of all structurally linked responses) in the various grammatical categories for Type 4 questions per participant**.

**Participant**	***S.T*. *n* = (36) 36**	***K.J*. *n* = (40) 40**	**P.I. *n* = (75) 47**	**K.M. *n* = (60) 58**	**S.H. *n* = (84) 70**
Simple situative statement	(66.67%) 66.67%	(20%) 20%	(100% 75)	(70% 42)	(52.38%) 62.86%
	24	8	100% 47	68.97% 40	44
Sentence with subjunctive mood	(8.33%) 8.33%	(20%) 20%	–	(30%) 31.03%	**–**
	3	8		18	
*That* + situative statement	(25%) 25%	–	–	–	(42.86% 36)
	9				31.43% 22
*That* + descriptiveclause		(60%) 60%	–	–	(4.76%) 5.71%
		24			4

*S.T. and K.J. are Wernicke's aphasics and P.I., K.M. and S.H. are Broca's aphasics*.

The strategy outlined above was successful especially for Broca's aphasics. A large majority of the grammatical responses produced by Broca's aphasics were situative statements containing “ToM” type reasoning. See Table 7.

**Table 7:** **Numbers and percentages of grammatical responses (in brackets: those of all structurally linked responses) in the various grammatical categories across aphasia types and in the control group for Type 4 questions**.

**Participant**	**Wernicke's aphasics *n* = (76) 76**	**Broca's aphasics *n* = (219) 175**	**Control group *n* = (982) 982**
Simple situative	(42.11%) 42.11%	(73.52% 161)	(30.96%) 30.96%
statement	32	74.86% 131	304
			
Sentence with	(14.47%) 14.47%	(8.22%) 10.29%	**–**
subjunctive mood	11	18	
			
*That* + situative	(11.84%) 11.84%	(16.44% 36)	(24.03%) 24.03%
statement	9	12.57% 22	236
			
*That* + descriptive	(31.58%) 31.58%	(1.83%) 2.29%	(44.83%) 44.83%
clause	24	4	442
			

The distribution of grammatical structures of structurally linked responses shows significant differences among aphasics and the control group [χ^2^-tests, Broca vs. control: $\chi_{\left(3,\;N=1157\right)}^{2}=256.23$, *p* < 0.001, *V* = 0.47, Wernicke vs. control: $\chi_{\left(3,\;N=1058\right)}^{2}$ = 152.31, *p* < 0.001, *V* = 0.38]. Also, aphasics produce significantly fewer recursive structures than the control group [χ^2^-tests, Broca vs. control: $\chi_{\left(2,\;N=1157\right)}^{2}$ = 183.05, *p* < 0.001, *V* = 0.4, Wernicke vs. control: $\chi_{\left(2,\;N=1058\right)}^{2}$ = 21.01, *p* < 0.001, *V* = 0.14]. As for grammatical situative statements, their frequency is significantly higher in Broca aphasics than in the control group but Wernicke aphasics do not differ significantly from the control group with respect to situative statements [χ^2^-tests, Broca vs. control: $\chi_{\left(2,\;N=1157\right)}^{2}$ = 65.07, *p* < 0.001, *V* = 0.24, Wernicke vs. control: $\chi_{\left(2,\;N=1058\right)}^{2}$ = 0.07, *p* > 0.05, *V* = 0.005].

## 4. Discussion

**4.1**. Recursive sentence embedding is impaired in Broca's aphasia. This is suggested by the fact thatmost of the Broca's aphasics' grammatical answers to Type 4 questions were simple situative statements, andonly very few were descriptive clauses introduced by a subordinating conjunction. The frequency of situative statements was significantly higher in Broca's aphasics than in the control group. On the other hand, Wernicke's aphasics did not differ significantly from the control group with respect to situative statements, and a few situative statementsbeginning with a subordinating conjunctionwere also produced. Both aphasic groups produced significantly fewer recursive structures than the control group, but recursive sentence embedding was less impaired in Wernicke's aphasia.

The use of simple situative sentences could also be observed in the case of the control group, but only in about a third of their responses. All other replies they gave were recursive structures, the answers contained syntactic subordination in overt forms: descriptive clauses or situative statements were intoduced by a subordinating conjunction. Therefore, recursive sentence embedding and ToM reasoning in the form of simple clauses represent two alternative strategies of which members of the control group were able to choose at will, whereas the aphasics were forced to choose the use of situative statements.

**Figure 6:**
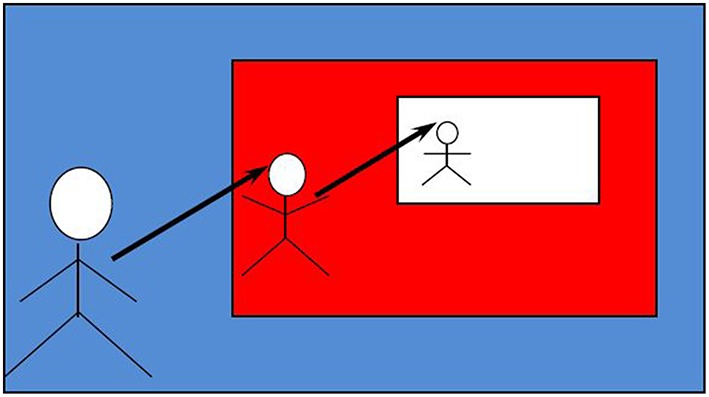
**The parallel between theory-of-mind embeddings and syntactic structural embeddings (Sauerland, 2005)**.

**4.2**. Bánréti (2010) was content with showing that aphasics tend to exploit the parallel between ToM reasoning and syntactic-structural embeddings (Sauerland, 2005) in order to avoid syntactic structural recursion in answering Type 4 questions. See Figure 6.

Now we wish to argue that more is at stake. The distribution of grammatical structures of structurally linked responses showed significant differences among aphasics and the control group. These results yield arguments supporting the claim that, along with impairments in recursive sentence embedding, recursive ToM inferences may remain selectively unimpaired in certain types of aphasia. By “recursion” in ToM inferences we mean that the participants, in addition to seeing themselves as able to infer other people's mental states, considered other persons (e.g., ones seen in pictures) to be able to infer further (third) persons' mental states, thus exhibiting recursive constructions. The content of situative statements showed that Broca's aphasic participants correctly identified themselves with the mental states of the characters in the pictures, thus complex syntactic structural recursion was avoided. Recursive sentence embedding was substituted for by simple clauses expressing ToM inferences. The subset of linguistic devices indicating non-descriptive perspective was available for the aphasic participants: ToM statements contain the first person singular feature (instead of the third person), the structures used were simple, sometimes fragmented correctly, their meaning referred to simple emotions, etc. Recursive sentence embedding, on the other hand, requires introductory formulas, subordinate conjunctions, agreement relations between main and embedded clauses, two propositions, etc. to control a descriptive perspective. This linguistic subsystem was only partially available or was not available for aphasic participants. Hence, in order to compensate for the deficiency, they resorted to another recursive subsystem.

## 5. Experiment #2: Participants with Alzheimer's disease

The inclusion of AD participants in our investigations was motivated by the fact that various studies have found patterns of linguistic deficits in AD. See the details in Section **1.4**. It has not been investigated so far how the linguistic deficits affect the operations of recursive clause embedding in AD. As far as we know, the relationship between AD persons' recursive sentence embedding abilities and their ToM reasoning abilities have not yet been explored systematically.

In persons with AD, as opposed to the case of aphasics, the language faculty becomes limited gradually due to a progression of microscopic neuropathological changes (Kempler, 2005; Hyman et al., 2012). We assumed that in a different type of linguistic impairment we would find a different distribution of responses. Thus, we administered the tests presented in Section Materials and Methods above to persons living with Alzheimer's disease.

### 5.1. Participants

The group of AD participants included 4 mild and 2 moderate AD participants. The native Hungarian speaking AD participants were categorized as mild vs. moderate based on the degree of their dementia with the help of the Mini Mental State Examination (MMSE) (Folstein et al., 1975; Tariska et al., 1990) and the ADAS-Cog test (Rosen et al., 1984). The participants met the diagnostic requirements of DSM-IV (American Psychiatric Association, 2000) and of ICD-10 (WHO, 1993) for AD. See some details in Table 8.

**Table 8:** **Data of the AD participants**.

**Participant**	**T.I**.	**To.Is**.	**Zs.A**.	**H.L**.	**K.F**.	**K.D**.	**Mean**
Age	75	78	55	63	72	75	69.67
Education	11	11	11	17	11	16	12.83
Sex	F	M	F	M	M	F	–
Handed	Right	Right	Right	Right	Right	Right	–
MMSE	24	20	25	24	15	15	20.5
Diagnosis	Mild AD	Mild AD	Mild AD	Mild AD	Moderate AD	Moderate AD	–

The healthy control participants, matched in age to the AD participants, are shown in Table 9.

**Table 9:** **Data of the control group**.

**Participant**	**F.Gy**.	**M.Zs**.	**Sz.E**.	**M.J**.	**K.J**.	**SZ.I**.	**Mean**
Age	71	55	66	78	71	62	67.17
Education	16	16	16	15	8	16	14.50
Sex	M	F	F	F	F	M	–
Handed	Right	Right	Right	Right	Right	Right	–
MMSE	29	30	30	29	29	30	29.5

### 5.2. Materials and methods

Medical/clinical tests as well as cognitive tests, including MMSE, were followed within 1 month by our own recursive sentence embedding tests administered in three sessions (three subtests), with at least 10 days elapsing between subsequent occasions.

We administered the above pictures and questions to AD participants. For stimuli, we used the same extended test material that was used with aphasic participants. Two hundred and eight photos depicting situations of everyday life were selected (Stark, 1998). We asked the four structural types of questions, ordered randomly. See the details in Section Materials and methods.

The research has been approved by the Research Ethics Committee of the Research Institute for Linguistics of the Hungarian Academy of Sciences, Budapest, Hungary (30/7/2014). All participants provided consent before participating in the test sessions.

## 6. Results

### 6.1. Some features of responses

**Figure 7:**
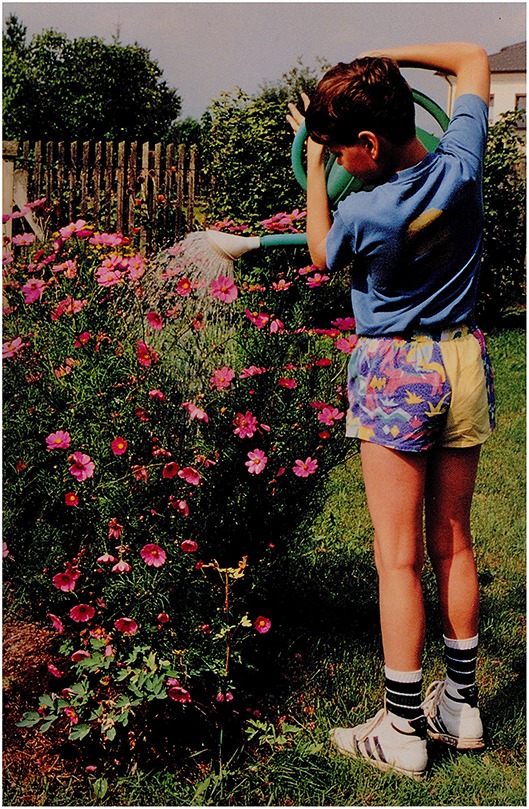
**A boy is watering flowers (Stark, 1998)**.

Fragments were avoided, and a preference for finite Verbs was followed. Infinitives were substituted for finite Verbs in the responses to Type 2 questions. Some specific attitude predicates requiring infinitive complements were avoided [*utál* “hate (to do something),” *szeret* “like (to do something),” *akar* “want (to do something),” or *legszórakoztatóbb*” most entertaining (to do),” etc.] and descriptive finite verbs were used instead of infinitives. See Figure 7:

The picture: A boy is watering flowers.

Question: Ő* mit utál?*

             ‘What does he hate to do?’

Answer by mild AD participant: *Hát locsol…virágot, rózsákat locsolják*.

          ‘Well, he is watering …flowers, roses are being watered.’

### 6.2. Examples for the grammatical types of responses to type 4 questions

**Figure 8:**
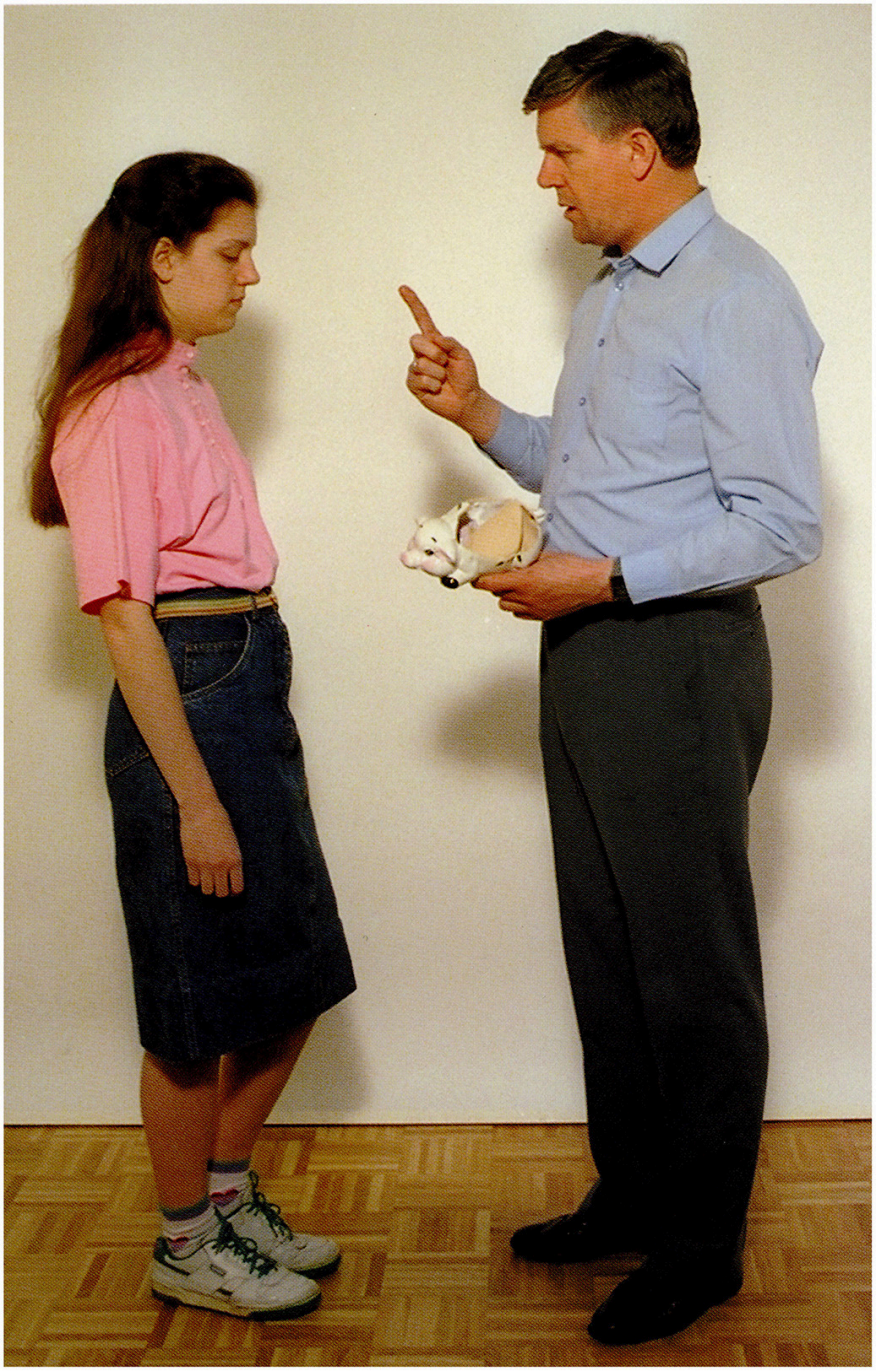
**A man scolds a girl (for breaking the piggy bank) (Stark, 1998)**.

**(a)** Simple clause describing intention: Figure 8.

The picture: A man scolds a girl (for breaking the piggy bank).

Question: *Mit mondhat a férfi a lánynak?*

             ‘What may the man be saying to the girl?’

Answer by mild AD participant: *Oktatja valamire a lányát*.

                   ‘He is teaching his daughter about something.’

**Figure 9:**
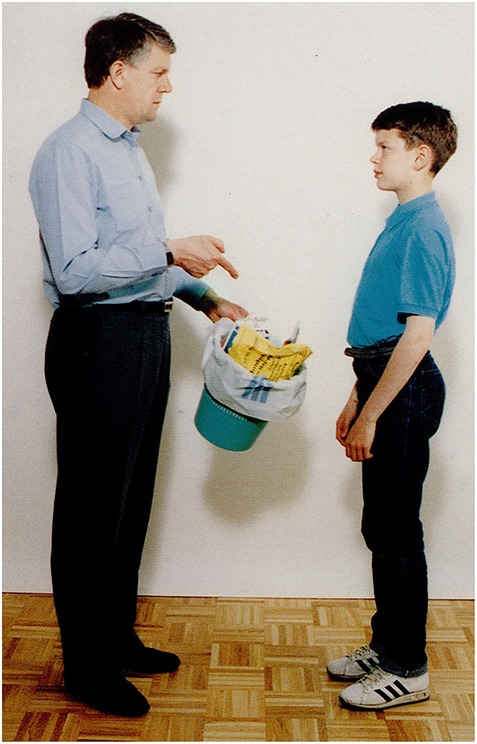
**A man orders a boy to take the garbage out (Stark, 1998)**.

**(b)** Simple descriptive clause with subjunctive mood (without *that*): Figure 9.

The picture: A man orders a boy to take the garbage out.

Question: *Mit mondhat az apa a fiúnak?*

             ‘What may the father be saying to the son?’

Answer by mild AD participant:*Vi****gye*** *ki a szemetet*.

                                   ‘He should take the garbage out.’

**Figure 10:**
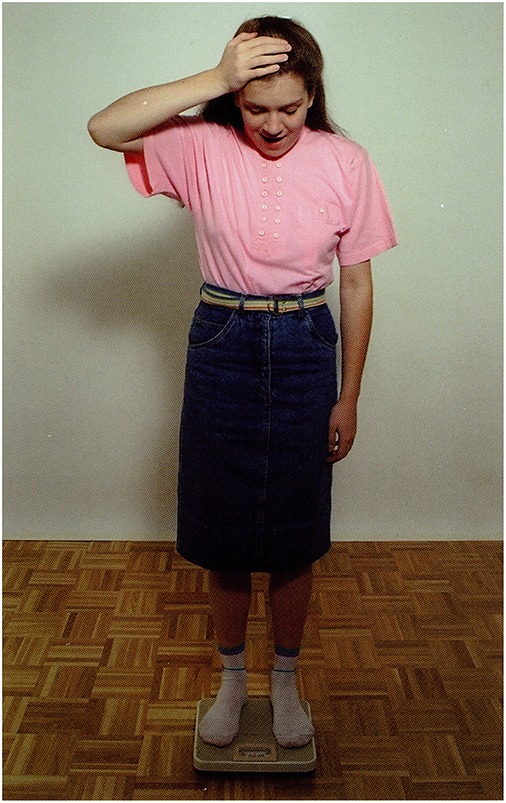
**A girl is standing on the bathroom scales (Stark, 1998)**.

**(c)** Descriptive clause with recursive embedding (*that*-clauses): Figure 10.

The picture: A girl is standing on the bathroom scales.

Question: *Vajon mire gondol a lány?*

             ‘What may the girl be thinking of?’

Answer by mild AD participant: *Arra, hogy megint hízott, vagy megint fogyott*.

             ‘That she put on weight again, or lost weight again.’

**Figure 11:**
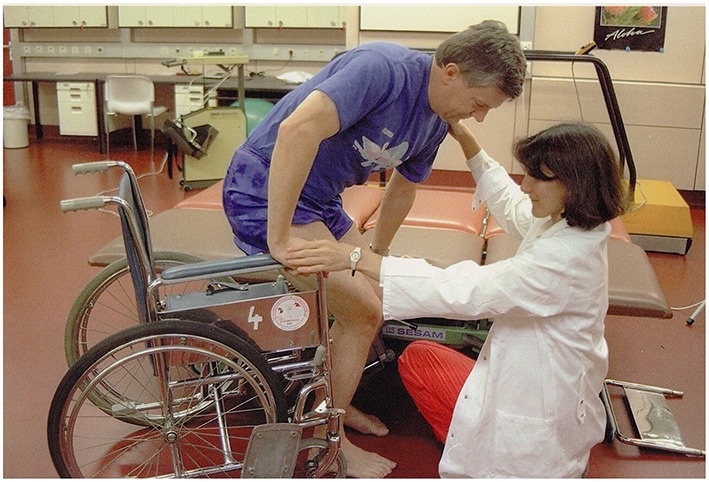
**A man is standing up from a wheelchair (Stark, 1998)**.

**(d)** Situative statement: Figure 11.

The picture: A man is standing up from a wheelchair.

Question: *Mire kérheti a férfi a nőt?*

             ‘What may the man be asking the woman to do?’

Answer by mild AD participant: *Segíts bele …a biciklibe!*

                         ‘Could you help me into …into the bike?’

### 6.3. Relevant and irrelevant responses: Examples

The responses by the AD participants were categorized from the point of view of relevance of the content as well. The relevance of the answer was evaluated in the context of the stimulus picture and the question heard.

**6.3.1**. Responses relevant in their content and fitting in structure: examples appear in Figures 7–11.

**Figure 12:**
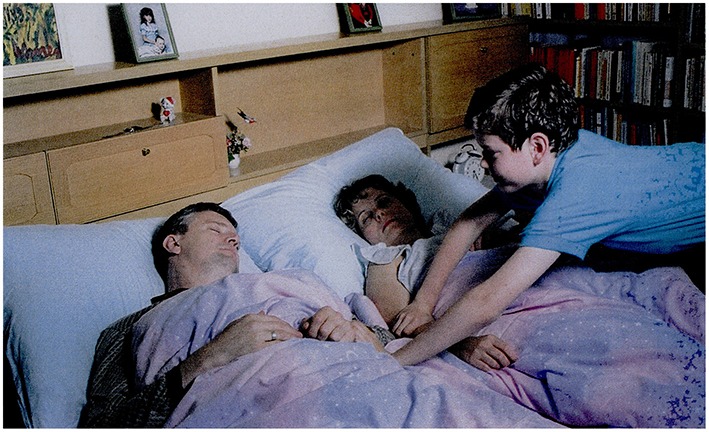
**A boy is waking up his father (Stark, 1998)**.

**6.3.2**. Responses not relevant in their content but fitting in structure: an example is given in Figure 12 below.

The picture: A boy wakes up his father.

Question: *Mi lehet a szándéka a fiúnak?*

             ‘What could be the intention of the boy?’

Answer by moderate AD participant: *El akar szőkni*.

                                                    ‘He wants to escape.’

**Figure 13:**
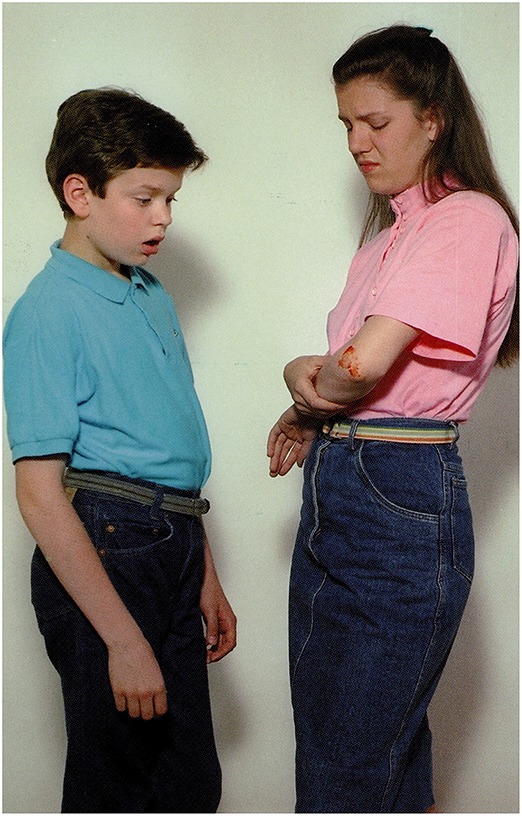
**A girl is showing her scar to a boy (Stark, 1998)**.

**6.3.3**. Syntactic structural recursion with incorrect content: Figure 13.

The picture: A girl is showing her scar to a boy.

Question: *Vajon mire gondol a fiú?*

             ‘What may the boy be thinking of?’

Moderate AD participant 1: *Hát, nem tudom hogy ő ezért., azt hogy ilyen nagyra akar nőni ő* is.

‘Well, I don't know that he therefore…that he wants to grow this big, too.’

Moderate AD participant 2:

*A fiú el van szomorodva, valami olyat mondott neki a lány, hogy elszomorodott, esetleg hogy nem szereti*.

‘The boy is sad, the girl told him something that made him sad, perhaps she said she did not love him.’

**Figure 14:**
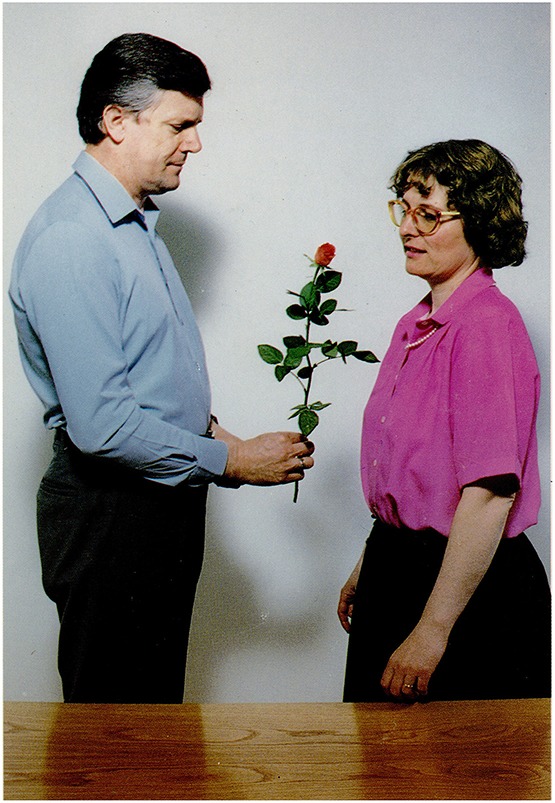
**A man is giving a flower to a woman (Stark, 1998)**.

**6.3.4**. Incorrect assignments of thematic roles: Figure 14.

The picture: A man is giving a flower to a woman.

Question: *Mire gondolhat a férfi?*

             ‘What may the man be thinking of?’

Answer by moderate AD participant: *Hogy milyen alkalomra* *kapta* *a virágot*.

                   ‘What kind of occasion he got the flower for.’

### 6.4. Grammaticality of responses by AD participants

Most of the responses by mild AD participants and moderate AD participants were grammatical, see Table 10.

**Table 10:** **Numbers and percentages of grammatical responses (in brackets: those of all structurally linked responses) for Type 1, 2, 3, and 4 questions per participant**.

**Participant**	**T.I. Mild**	**To.Is. Mild**	**Zs.A. Mild**	**H.L. Mild**	**K.F. Moderate**	**K.D. Moderate**
Type **1**	97.87%	98%	98%	100%	97.5%	100%
question	(47) 46	(50) 49	(50) 49	(52) 52	(40) 39	(40) 40
Type **2**	97.37%	98.04%	100%	100%	100%	100%
question	(38) 37	(51) 50	(38) 38	(52) 52	(34) 34	(40) 40
Type **3**	100%	100%	100%	100%	100%	100%
question	(39) 39	(51) 51	(52) 52	(52) 52	(40) 40	(40) 40
Type **4**	100%	100%	100%	100%	96.36%	95.45%
question	(31) 31	(30) 30	(23) 23	(22) 22	(55) 53	(44) 42

*Mild AD participants: T.I., To.Is., Zs.A., H.I., moderate AD participants: K.F., K.D*.

As Table 10 shows, most of the responses to Type 1–4 questions were grammatical.

### 6.5. Responses to type 4 questions: Mild AD participants

Responses by mild AD participants to Type 4 questions in terms of grammatical categories are shown in Table 11.

**Table 11:** **Numbers and percentages of grammatical responses by mild AD participants and the control group to Type 4 questions in terms of grammatical categories**.

**Participant**	**T.I. *n* = 31**	**To.Is. *n* = 30**	**Zs.A. *n* = 23**	**H.L. *n* = 22**	**Mild AD group: total *n* = 106**	**Control group: *n* = 114**
**NON-RECURSIVE**						
Simple descriptive sentence	22.58%	6.67%	21.74%	4.55%	14.15% 15	14.91% 17
	7	2	5	1		
Simple sentence with subjunctive	**–**	**–**	13.04%	**–**	2.83% 3	**–**
			3			
Simple situative statement	**–**	20%	39.13%	27.27%	19.81% 21	30.7% 35
		6	9	6		
Total for non-recursive structures	22.58%	26.27%	73.91%	31.82%	36.79% 39	45.61% 52
	7	8	17	7		
**RECURSIVE STRUCTURES**						
*That* + situative statement	**–**	**–**	**–**	27.27%	5.66% 6	6.14% 7
				6		
*That* + descriptive clause	41.94%	60%	13.04%	22.73% 5	36.79% 39	40.35% 46
	13	18	3			
*That* + clause with subjunctive	35.48%	13.33%	13.04%	18.18%	20.75% 22	7.89% 9
	11	4	3	4		
Total for *that*-clauses	77.42%	73.33%	26.08%	68.18%	63.21% 67	54.39% 62
	24	22	6	15		
Total for situative statements	**–**	20%	39.13%	54.55%	25.47% 27	36.84% 42
		6	9	12		
Total for non-situative statements	100%	24% 24	60.87%	45.45%	74.53% 79	63.16% 72
	31		14	10		

Comparing the distribution of recursive responses to Type 4 questions, statistical analysis shows that there is no significant difference between the group of mild AD participants and the control group [χ^2^-test, $\chi_{\left(2,\;N=220\right)}^{2}$ = 1.76, *p* > 0.05, *V* = 0.09]. The same is true for situative and non-situative statements [χ^2^-test, $\chi_{\left(3,\;N=220\right)}^{2}$ = 3.3, *p* > 0.05, *V* = 0.12]. The results suggest that in mild AD participants recursive sentence embedding is not affected. As to individual members of the AD group, the pattern of Zs.A.'s responses was different: she produced fewer *that*-clauses and more situative statements than other mild AD participants did in responses to Type 4 questions.

### 6.6. Responses to type 4 questions: Moderate AD participants

The number of responses by moderate AD participants to Type 4 questions in various grammatical categories is shown in Table 12.

**Table 12:** **Numbers and percentages of grammatical responses by moderate AD participants and the control group to Type 4 questions in terms of grammatical categories**.

**Participant**	**K.F. *n* = 53**	**K.D. *n* = 42**	**Moderate AD group total *n* = 95**	**Control group mean *n* = 114**
**NON-RECURSIVE**				
Simple descriptive sentence	18.86% 10	23.81% 10	21.05% 20	14.91% 17
Simple sentence in subjunctive	26.42% 14	7.14% 3	17.89% 17	**–**
Situative statement	3.77% 2	**–**	2.11% 2	30.7% 35
Total for grammatical non-recursive structures	49.06% 26	30.95% 13	41.05% 39	45.61% 52
**RECURSIVE STRUCTURES**				
*That* + situative statement	**–**	4.76% 2	2.11% 2	6.14% 7
*That* + descriptive clause	50.94% 27	64.29% 27	56.84% 54	40.35% 46
*That* + clause with subjunctive	**–**	**–**	**–**	7.89% 9
Total for grammatical *that*-clauses:	50.94% 27	69.05% 29	58.94% 56	54.39% 62
Total for grammatical situative statements	3.78% 2	4.76% 2	4.21% 4	36.84% 42
Total for grammatical non-situative statements	96.23% 51	95.24% 40	95.79% 91	63.16% 72

The data show that there is significant difference in the proportion of situative statement responses between the moderate AD group and the control group [χ^2^-test, $\chi_{\left(2,\;N=209\right)}^{2}$ = 32.14, *p* < 0.001, *V* = 0.39]. On the other hand, there is **no** significant difference in the distribution of grammatical recursive–non-recursive structures [χ^2^-test, $\chi_{\left(2,\;N=209\right)}^{2}$ = 0.44, *p* > 0.05, *V* = 0.05], which suggests that in moderate AD participants syntactic-structural recursion is not affected.

### 6.7. Relevance of the content in responses to type 4 questions

The relevance of the answer was evaluated in the context of the stimulus picture and the question heard (cf. Figures 12–14). In a relevant response participant answered the question and talked about the picture, in a non-relevant response participant did not answer the question and/or did not talk about the picture. The performance of mild and moderate AD participants is shown in Table 13.

**Table 13:** **Relevance of the content in the responses to Type 4 questions by mild and moderate AD participants: numbers and percentages of relevant and irrelevant responses**.

**Participant**	**H.L. (mild) *n* = 22**	**To.Is. (mild) *n* = 30**	**T.I. (mild) *n* = 31**	**Zs.A. (mild) *n* = 23**	**K.F. (moderate) *n* = 55**	**K.D. (moderate) *n* = 44**
Relevant responses	86.36%	76.67%	70.97%	82.61%	56.36%	65.91%
(The participant answered the question and talked about the picture)	19	23	22	19	31	29
Non-relevant responses	13.64% 3	23.33%	29.03%	17.39%	43.64%	34.09%
(The participant did not answer the question and/or did not talk about the picture)		7	9	4	24	15

Table 13 shows that the number of irrelevant responses to Type 4 questions is significantly higher in moderate AD participants [χ^2^-test, $\chi_{\left(2,\;N=205\right)}^{2}$ = 7.6, *p* < 0.01, *V* = 0.19].

## 7. Summary of results

### 7.1. Recursive sentence embedding

The share of *that*-clauses jumped up in the responses to Type 4 questions for all AD groups. The ratio of recursive sentence embedding is considerably greater in the case of Type 4 questions than in the case of Type 1, 2 and 3 questions: see Table 14. The distribution of **grammatical** recursive structures shows significant differences among question types in the case of both types of AD [χ^2^-tests, mild AD: $\chi_{\left(3,\;N=688\right)}^{2}$ = 230.02, *p* < 0.001, *V* = 0.58, moderate AD: $\chi_{\left(3,\;N=333\right)}^{2}$ = 116.97, *p* < 0.001, *V* = 0.59], Table 14.

**Table 14:** **Numbers and percentages of grammatically well-formed recursive (R) vs. non-recursive (NR) answers by mild vs. moderate AD participants vs. the control group in Type 1–4 questions**.

**Question**	**Mild AD: 4 participants**		**Moderate AD: 2 participants**		**Control group: 6 participants**	
	**Recursive**	**Non-recursive**	**Recursive**	**Non-recursive**	**Recursive**	**Non-recursive**
Type **1** question	1% 2	99% 197	1.25% 1	98.75% 79	0% 0	100% 323
Type **2** question	6.7% 12	93.3% 167	2.7% 2	97.3% 72	3.02% 10	96.97% 321
Type **3** question	12.37% 24	87.63% 170	12.5% 10	87.5% 70	14.62% 50	85.38% 292
Type **4** question	63.21% 67	36.79% 39	58.95% 56	41.05% 39	54.39% 62	45.61% 52

### 7.2. Relevance of the content in responses to type 4 questions

Relevant and irrelevant responses to Type 4 questions: a total for the AD groups is shown in Table 15.

**Table 15:** **Relevant and irrelevant responses to Type 4 questions**.

**Participant**	**Mild AD *n* = 106**	**Moderate AD *n* = 99**
Relevant responses	78.3%	60.6%
(The participant answered the question and talked about the picture)	83	60
Irrelevant responses	21.7%	39.4%
(The participant did not answer the question and/or did not talk about the picture)	23	39

### 7.3. Grammatical categories of responses to type 4 questions

Within all responses given by moderate AD participants the ratio of situative statements (4.21%) was far lower than the ratio of situative statements (25.47%) within all responses given by persons with mild AD. In this respect, mild AD participants behave similarly to healthy controls (i.e., there is no significant difference in the distribution of situative and non-situative statements for either group) but moderate AD participants produce significantly fewer situative statements than either the mild AD or the control group (see Tables 11, 12, 14).

We also found that mild AD participants tend to produce a high number of situative statements and a low number of irrelevant answers whereas moderate AD participants tend to produce a low number of situative statements and a high number of irrelevant answers (results are not significant, Pearson's correlation, *r*_(6)_ = −0.6927, *p* > 0.05). See Tables 11–13, 15.

## 8. An additional experiment: The two moderate AD participants

In our tests exploring recursive clause embedding abilities, the grammatical responses given by Broca's and Wernicke's aphasics did not contain any instance of wrong ToM reasoning. On the other hand, the number of instances of false ToM reasoning occurring in responses given by the moderate AD group was significantly higher than in responses given by mild AD participants. So, in the case of moderate AD participants, we used another kind of test to see if this was a task specific effect—for instance, whether a potential limitation of the visual perception system in moderate AD caused the ToM deficit appearing in linguistic responses (the participants had to answer questions concerning photographs they were looking at)—or if the ToM limitation shows up across task types in moderate AD. We therefore administered first and second-order false belief tests to our two moderate AD subjects.

### 8.1. Participants

The two native Hungarian speaking moderate AD participants were involved. They were categorized as moderate based on the degree of their dementia with the help of the MMSE (Folstein et al., 1975; Tariska et al., 1990) and the ADAS-Cog test (Rosen et al., 1984). The participants met the diagnostic requirements of DSM-IV (American Psychiatric Association, 2000) and of ICD-10 (WHO, 1993) for AD. See some details in Table 8 above.

### 8.2. Materials and design

We used a first-order (six sentences long) and a second-order (eight sentences long) false belief test following Youmans and Bourgeois (2010). In this experiment we read out two stories to the participants, while they were able to follow it also from the text which we handed over to them, following these instructions:

“*Here is a very short story I want to give you. I will read the story out loud. Please, read this same story from this text to yourself while I read it out. Please, make sure you pay close attention to it, because when I'm done reading it, I will ask you a few questions about the story, which you should remember. Do you have any questions?”*

We asked four questions in connection with each story, which we also handed over in writing to the moderate AD participants. The questions focused on the following: 1. false beliefs, 2. comprehension, 3. remembering, 4. general conclusions.

The research has been approved by the Research Ethics Committee of the Research Institute for Linguistics of the Hungarian Academy of Sciences, Budapest, Hungary (30/7/2014). All participants provided consent before participating in the test sessions.

### 8.3. Results

#### 8.3.1. First-order false belief story

*John and his wife Margaret arrive home. They park their car in the driveway in front of their house. Margaret goes upstairs to take a shower. After Margaret goes upstairs, John decides that it is going to rain. John moves the car into the garage. Later, Margaret remembers she is out of milk, and decides to drive to the grocery store* (Youmans and Bourgeois, 2010; Hoffmann et al., 2011)

False belief: Where will Margaret look first for the car? (Expected response: driveway, outside, in front of house)

K.F.'s response: *driveway* K.D.'s response: *outside, driveway*

Comprehension: Where is the car at the end of the story? (Expected response: in the garage)

K.F.'s response: *in the garage* K.D.'s response: *in the garage*

Memory: Where was the car parked at the beginning of the story? (Expected response: driveway, in front of house)

K.F.'s response: *driveway* K.D.'s response: *driveway, outside*

Physical inference: If it rains, will it rain on the car? (Expected response: no)

K.F.'s response: *no* K.D.'s response: *not, it will not*

#### 8.3.2. Second-order false belief story

*Mary wants to hide Peter's birthday present. She wants to trick Peter, so he won't be able to find his present. Mary says, “Peter, close your eyes, I'm going to hide your present here in the living room.” When Peter closes his eyes, Mary runs quietly up the stairs. At the top of the stairs Mary knocks some dirt out of a potted plant. Upstairs, Mary goes into the bedroom to hide Peter's present. But Peter peeked! He saw Mary climb the stairs and go into the bedroom with his present* (Youmans and Bourgeois, 2010; Hoffmann et al., 2011).

False belief: Where does Mary think that Peter thinks his present is hidden? (Expected response: in the living room. The incorrect answers are marked in red.)

False belief: Where does Mary think that Peter thinks his present is hidden? (Expected response: in the living room. The incorrect answers are marked in red.)

K.F.'s response: ^*^ upstairs K.D.'s response: ^*^ in the bedroom

Comprehension: Where is the present hidden? (Expected response: in the bedroom, upstairs.)

K.F.'s response: *upstairs* K.D.'s response: *in the bedroom*

Memory: Where does Mary tell Peter she is hiding his present? (Expected response: in the living room, downstairs.)

K.F.'s response: *well, in the living room* K.D.'s response: *in the living room*

Physical inference: Where would there be spilled dirt? (Expected response: top of stairs, on the stairs, upstairs.)

K.F.'s response: *upstairs* K.D.'s response: *top of stairs*

The first-order false belief test did not present any difficulty to the participants, who provided correct answers to all the questions. The second-order false belief test turned out to be more difficult for the two moderate AD participants. Despite the fact that the participants were assisted in their remembering (in having the text of the story in front of them), **both of them** provided incorrect answers to false belief questions.

## 9. Discussion

There was no significant difference between the mild AD group and the control group in the proportions of replies involving recursive sentence embedding vs. situative responses. We can infer that in mild AD, syntactic-structural recursion is unaffected.

In the case of moderate AD group we found a significant difference in the proportion of responses involving recursive sentence embedding and simple situative statements: the ratio of situational statements presupposing recursive ToM reasoning was significantly lower than in the control group. The moderate AD group produced significantly fewer situative statements than the healthy control participants did [χ^2^-test, $\chi_{\left(2,\;N=209\right)}^{2}$ = 32.14, *p* < 0.001, *V* = 0.39]. On the other hand, the share of sentences involving syntactic-structural recursion (*hogy* “that”-clauses embedded) was not lower than in healthy control responses. Additionally, we also received semantically irrelevant responses: exhibiting irrelevant situative statements or referring to some irrelevant parts of the picture. This finding demonstrates that while recursive sentence embedding is unimpaired in moderate AD, the recursive ToM reasoning can be limited.

We found that the second-order false belief test proved to be difficult for the two moderate AD participants: they gave the wrong answers. Their performance makes it probable that the ToM deficit occurring in their responses given in clause embedding tests was not exclusively a task specific effect, given that ToM deficit showed up in another type of task, too, with the same two moderate AD persons.

## 10. General discussion

### 10.1. Double dissociation

In mild and moderate AD, abilities to use recursive sentence embedding (with *that*-clauses embedded) remain unaffected. ToM inferences become limited by the moderate stage of the disease. Moderate AD participants tend to avoid simple situative statements (that assume the state of mind of another person). The share of irrelevent responses jumped up in the responses to Type 4 questions by moderate AD participants. Unlimited subsystem of recursive sentence embedding and limited ToM inferences in moderate AD: this result exhibits a pattern of dissociation.

In the case of Broca's aphasics, limited subsystem of recursive sentence embedding but unimpaired ToM inferences were found. Recursive sentence embedding was substituted for by simple clauses expressing ToM inferences.

Thus, we found double dissociation. Limited subsystem of recursive sentence embedding and unimpaired ToM inferences in Broca's aphasia as opposed to unlimited recursive sentence embedding and limited ToM inferences in AD: this is a pattern of double dissociation. One the one hand, this finding supports theories (e.g., Zimmerer and Varley, 2010) claiming that the specific recursive rules are distinct in adults. On the other hand, the above-sketched double dissociation may yield a relevant problem for theories claiming that recursive sentence embedding is based on ToM recursion. If this were the case, ToM limitations should have caused syntactic disturbances in the moderate AD group, yet we did not find any.

10.2. The dissociation of syntactic-structural recursion and ToM inferences can be observed in Wernicke's aphasia to a lesser degree; this is in harmony with earlier observations that associate limited syntactic abilities primarily with Broca's aphasia and consider grammatical errors committed by Wernicke's aphasics as consequences of the impairment of some lexical processes.

### 10.3. The emergence of a compensatory strategy

The Broca's aphasic participants use recursive ToM inferences (in situative statements of simple syntactic structure) in exactly the cases where the structure of Type 4 questions would have potentially supported answers with recursive sentence embedding. By using this compensation strategy, they successfully resolved the task. Therefore, the dissociated recursive subsystems are not totally independent of one another: the impairment of one may trigger the use of another one as a substitution mechanism or repair strategy; they are utilizable in strategies such that “one may replace the other.”

We found that operations of recursive sentence embedding remained intact in mild and moderate AD. On the other hand, in the moderate phase of the disease, the AD participants gave wrong answers with respect to other people's intentions and avoided situative statements carrying ToM inferences. Retained syntactic abilities of this group failed to offer any compensation strategy to help them solve the tasks in the test. There was no linguistic substitution mechanism or repair strategy available for them.

### 10.4. Compensatory strategy: The “two systems” approach

Our participants were given complex tasks in the test. Photographs of real life situations were presented and various types of questions were asked about them. Participants had to respond to questions by judging the situation seen in the photo. This required their using complex systems in complex cognitive domains.

Some researchers propose a dual process theory for operations in complex cognitive domains. For the neural base of recursion and computing hierarchical structures, Friederici et al. (2011) propose that there are two parallel computational systems processing hierarchical structures in the lateral prefrontal cortex. One system which follows the posterior-to-anterior gradient is determined by cognitive control. This is a less automatic system processing complex sequences in different domains, among others in some non-language domains and giving rise to activation in the anterior prefrontal cortex (BA 47/45a and 10). Another system processing the recursive syntactic hierarchy of natural language activates the more posterior regions of the inferior frontal gyrus, covering Broca's area. This system is highly automatic in adults. “Language processing in adults is highly automatic and does not appear to be very challenging for the brain, even when the sequences to be processed are hierarchically complex […] humans are predetermined to compute linguistic recursion, with BA 44/45p being the neural correlate of this showing its functional primacy in adult brain” (Friederici et al., 2011, p. 101).

The two systems that process hierarchical structures differ in their specific domain and the degree of cognitive control; namely, the first system is less automatic, while the second system is highly automatic.

The two systems approach is also proposed in the study of ToM (Apperly and Butterfill, 2009; Cohen and German, 2009; Strickland et al., 2014). According to Apperly and Butterfill, 2009), ToM operations involve at least two types of system. One type of system is quick, highly automatic, cognitively efficient, but limited and inflexible. This early-developing system has a central role in guiding online social interaction and simple everyday communication. Another type of system is highly flexible but cognitively inefficient, and requires more cognitive control. This later-developing flexible cognitive system is necessary for solving false-belief tasks and enable adults to perform explicit ToM reasoning in complex social interaction. For instance, it “enable[s] adults to engage in top-down guidance of social interaction (such as anticipating what the audience of a lecture might know or working out how one misjudged the audience afterward) and in explicit reasoning about the causes and justification of mental states (as in everyday practical reasoning or jurisprudence)” (Apperly and Butterfill, 2009, p. 966). The two separate systems co-exist in adults.

Strickland et al. (2014) presented studies on the link between syntax and intentionality judgements. They found two types of system in the intentionality judgements. A fast-acting reasoning system for automatically calculating others' mental states was found. This system acts on syntactic-structural information showing an automatic link between on-line language processing and ToM. When a more complicated design was employed in the experiments they found another way to generate intentionality judgements: this was a slower, deliberate, non-automatic system that was based on a deeper, more reflective understanding of the real-world events that particular sentences refer to. The syntactic biases did not affect intentionality judgements or influenced them less in this second case, and the event referred to by the sentence was considered in a deeper, reflective manner. According to Strickland et al. (2014), one possible way to interpret their results is the “two systems” framework.

10.5. We assume that the “two systems” approach can be applied in the interpretation of compensatory strategies shown by aphasic participants. In this case, the relevant two systems differ in their specific tasks, domain, and in the degree of automation. Recursively embedded clauses may trigger highly automatic computational operations in healthy participants. This system is impaired in Broca's aphasia. On the other hand, to solve the tasks in our tests, the participants also needed to perform a complex ToM reasoning in a type of recursive “logic” that was unimpaired and they used less automatic and more reflexive operations requiring more cognitive control. The complexity of the tasks in our experiment (participants had to judge a real-life situation shown in a picture and respond to a specific question they heard concerning the situation), compounded by impairments in recursively embedded clauses, triggered the application of compensatory strategies.

Broca's aphasics understood the questions on the mental states of the characters seen in the pictures (they did not give any irrelevant situative statement response). They were less impaired in sentence comprehension; they might realize potential recursion in the meaning of the question and the potential answer. On the other hand, the proper linguistic production side was not available for them. Their repair strategies were based on their knowledge of social contexts and their pragmatic aspects, as well as their accessible ToM system. In ToM responses, a comprehensive semantic and morphological use of the feature “first person” was discernible. Broca's aphasics, using their own minds as models, carried out a simulation and analysis of the mental states of persons seen in the test picture, and selected their conclusions from among those states. In the content of the responses, activation of a simulation-based mindreading (Gallese and Goldman, 1998; Goldman, 2006, 2012) was apparent, and the net result was implemented in brief, simple, non-recursive linguistic forms. In answering the questions of the test (*What may X be saying/thinking/reminding Y of/asking Y to do in the picture*), the participants offered situative statements that contained a simulation and analysis of the social situations, facial expressions, and emotions shown in the pictures, and the selected conclusions based on them. These results support the simulation model of mindreading (Gallese and Goldman, 1998; Goldman, 2006, 2012). Such working of the ToM system required significant cognitive control, too.

The above compensation strategy involves a switch from the impaired, highly automatic system to the unimpaired, non-automatic or less automatic system. Broca's aphasics employed highly reflexive ToM reasoning that required significant cognitive control in the framework of recursive “logic.” That strategy made it possible for them to solve the tasks in the test.

In moderate AD participants we found a reverse construction, in that automatic operations of recursive sentence embedding were well-functioning, there was no need for a repair strategy in this respect. Moderate AD participants successfully processed the meaning of the questions on the mental states of the characters seen in the picture. Linguistic questions were understood. But they were often unable to select, identify and interpret the relevant parts of the event seen in the picture from the point of view of the mental state of the characters seen in the picture. Some visually real but non-relevant parts of the photos were mentioned that had no connections to the essence of the situation and intentions of the characters in the photos (cf. Figure 13, for instance). The less automatic, reflexive ToM reasoning was impaired in moderate AD participants (This is compatible with the data suggesting that our two moderate AD participants exhibited poor performance in a second-order false belief test). Persons with moderate Alzheimer's disease had no linguistic mechanism at their disposal to compensate for that deficiency even though they were otherwise quite able to produce syntactically complex sentences.

10.6. The production differences between aphasic and AD participants observed in our tests are explained by the fact that we do not seem to have to do with a general recursive operation whose application may be impaired or remain intact at various levels; rather, we encounter separate recursive operations bound to subsystems that may be selectively impaired. In this case, the recursive operations are subsystem-specific. However, these operations are not independent of one another; this is shown by the available compensatory strategy. We have found that, whenever one of the recursive operations bound to a subsystem is impaired, another set of operations of another subsystem enters the scene, also involving recursion, as part of compensatory strategy. The main point of compensatory strategies followed by aphasics was a kind of change from an impaired automatic linguistic system to an unimpaired non-automatic ToM system.

## 11. Conclusions

The **double dissociation** of recursive sentence embedding and ToM recursion between the group of Broca's aphasics and people with moderate AD may pose relevant problems for theories claiming that recursive sentence embedding is based on ToM recursion. In the moderate AD group recursive sentence embedding was almost totally unimpaired while ToM inferences had severe limitations. These limitations did not cause any syntactic disturbances in the moderate AD group.

Broca's aphasics arrived at the right ToM inferences. They creatively hit upon the simple non-recursive linguistic form and the corresponding ToM **perspective** using “first person singular” reference to an imagined “ego” of the person in the picture as simulated by the aphasic participant whose combination made it possible to express recursive inferences in a non-recursive linguistic form. The change from third to first person represented a kind of perspective embedding by Broca's participants. A deficit in the subsystem of recursive sentence embedding was compensated for by perspective embedding in the ToM system. In this connection, our results support the simulation model of mind reading.

In Broca's aphasia and moderate AD, we have found separate recursive operations bound to subsystems. The nature of their interrelations makes it possible for one subsystem that remained unimpaired to compensate for the other's limitations in Broca's aphasia.

## Author contributions

All authors listed, have made substantial, direct and intellectual contribution to the work, and approved it for publication.

### Conflict of interest statement

The authors declare that the research was conducted in the absence of any commercial or financial relationships that could be construed as a potential conflict of interest. The reviewer CH and handling Editor declared their shared affiliation, and the handling Editor states that the process nevertheless met the standards of a fair and objective review.
